# Effects of AAV-mediated knockdown of nNOS and GPx-1 gene expression in rat hippocampus after traumatic brain injury

**DOI:** 10.1371/journal.pone.0185943

**Published:** 2017-10-10

**Authors:** Deborah R. Boone, Jeanna M. Leek, Michael T. Falduto, Karen E. O. Torres, Stacy L. Sell, Margaret A. Parsley, Jeremy C. Cowart, Tatsuo Uchida, Maria-Adelaide Micci, Douglas S. DeWitt, Donald S. Prough, Helen L. Hellmich

**Affiliations:** 1 Department of Anesthesiology, University of Texas Medical Branch, Galveston, Texas, United States of America; 2 GenUs Biosystems, Northbrook, Illinois, United States of America; University of South Florida, UNITED STATES

## Abstract

Virally mediated RNA interference (RNAi) to knock down injury-induced genes could improve functional outcome after traumatic brain injury (TBI); however, little is known about the consequences of gene knockdown on downstream cell signaling pathways and how RNAi influences neurodegeneration and behavior. Here, we assessed the effects of adeno-associated virus (AAV) siRNA vectors that target two genes with opposing roles in TBI pathogenesis: the allegedly detrimental neuronal nitric oxide synthase (nNOS) and the potentially protective glutathione peroxidase 1 (GPx-1). In rat hippocampal progenitor cells, three siRNAs that target different regions of each gene (nNOS, GPx-1) effectively knocked down gene expression. However, *in vivo*, in our rat model of fluid percussion brain injury, the consequences of AAV-siRNA were variable. One nNOS siRNA vector significantly reduced the number of degenerating hippocampal neurons and showed a tendency to improve working memory. GPx-1 siRNA treatment did not alter TBI-induced neurodegeneration or working memory deficits. Nevertheless, microarray analysis of laser captured, virus-infected neurons showed that knockdown of nNOS or GPx-1 was specific and had broad effects on downstream genes. Since nNOS knockdown only modestly ameliorated TBI-induced working memory deficits, despite widespread genomic changes, manipulating expression levels of single genes may not be sufficient to alter functional outcome after TBI.

## Introduction

Although acute and chronic neurodegeneration and long term deficits in cognitive function are hallmarks of traumatic brain injury (TBI) survivors, there are currently no treatments to address these debilitating repercussions [1–3]. Thus, new therapeutic modalities to restore normal brain function are worth exploring, such as genomic engineering strategies [4]. We and others have shown that TBI-induced changes in many genes may be causally linked to neurodegeneration [5–8] and that these injury-induced changes are linked to cognitive dysfunction [3]; however, little is known about the effects of manipulating gene expression levels on functional outcome post-TBI.

Disruption or knockdown of gene function by RNA interference (RNAi) has been explored because of its ubiquitous functionality in all organisms [9]. Remarkably, RNAi appears to work even across animal kingdoms, such as in plants and fungi [10]. Functional RNAi pathways are essential for development and cellular functions in mammalian cells. For example, in mice, knockout of the endoribonuclease Dicer, which is necessary for cleavage of double-stranded RNA into small interfering RNAs and microRNAs, results in an embryonic lethal phenotype [11]. Introducing RNAi vectors in the brain to inhibit target genes, rather than non-biological compounds, will effectively commandeer endogenous gene silencing machinery. It is well known that pharmacotherapeutic methods employed to repair or restore TBI-dysregulated gene expression have not lived up to their initial potential in preclinical studies and have failed to translate into treatments [12]. Thus, gene silencing or knockdown of deleterious genes using RNAi has been explored in recent years as a viable treatment option [13]. In 2003, Hommel et al. [14] reported that virally mediated RNAi in the midbrain of adult mice of tyrosine hydroxylase, an enzyme essential for synthesis of dopamine, could alter neurobehavioral phenotypes. This result suggests *in vivo* gene silencing via RNAi can improve functional outcome after TBI and can illuminate how a gene regulates specific brain functions, including behavior [15]. Subsequently, the possibility of RNAi for disease-modifying therapy has led to gene knockdown studies in several animal models of human neurodegenerative diseases, including brain injury [16–21]. Viral vectors used in these studies, such as adeno-associated virus (AAV), enable long-term gene knockdown but pose challenges that must be addressed. In moving forward with these *in vivo* genetic manipulation studies, it will be important to assess both pros and cons of prolonged gene silencing [22]. In particular, it will be necessary to determine whether AAV induced immune responses affect siRNA expression in the host tissues [23], although among all gene therapy vectors, AAV has been shown to be the least immunogenic [24].

Nitric oxide synthases (NOS) are injury-induced genes proposed as therapeutic targets for brain injury [25]. Neuronal nitric oxide synthase (nNOS) is widely and constitutively expressed in the brain and has essential physiological functions including synaptic signaling, neurovascular coupling [26–28], counteracting inflammatory signals in endothelial cells [29], and regulating the differentiation of neural progenitor cells (NPCs) [27]; however, increased levels of nNOS after TBI leads to oxidative-stress induced brain damage [30]. Moreover, nNOS is a good candidate for therapeutic knockdown because its increase is linked to neuronal death via poly (ADP-ribose) polymerase (PARP) activation [31]. In ischemic brain injury, a previous study showed disruption of nNOS signaling was potentially therapeutic [32]. Although lentiviral mediated RNAi was used to knockdown nNOS in rat piriform cortex, the functional consequences of gene silencing in this study were not reported [33]. In the present study, we investigated the hypothesis that knocking down nNOS will reduce neurodegeneration and improve hippocampal-dependent memory deficits after TBI.

The injured brain responds by activating endogenous reparative processes to counter neurodegeneration or remodel the brain to enhance functional recovery. Antioxidant genes, such as glutathione peroxidase, are essential for recovery of mitochondrial function after brain injury [34]. They are known to mediate neuroprotection in ischemic preconditioning [35] and are involved in suppression of neurodegeneration by PGC-1 transcriptional coactivators [36]. Glutathione peroxidase-1 (GPx-1) is a potent member of cell antioxidants upregulated in response to oxidative stress caused by TBI. Thus, we hypothesized that knockdown of GPx-1 will increase neurodegeneration and worsen functional outcome after TBI.

Here, we report that AAV-mediated RNAi, targeted towards two genes with postulated opposing roles in TBI pathogenesis, yielded both promising and confounding results in a rat TBI model. In addition to reducing neuronal injury, nNOS siRNA treatment resulted in wide scale genomic changes in multiple canonical signaling pathways associated with cell survival and neuronal functions. There were also modest trends toward improved working memory deficits in TBI rats. In contrast, we found that transiently knocking down GPx-1 in hippocampal neurons neither increased neuronal injury nor worsened working memory after TBI, despite affecting multiple cell signaling pathways. It may therefore be necessary to target more than one critical gene involved in cell death or survival to alter cognitive function.

## Materials and methods

### Ethics statement

All animal experiments were approved by the Institutional Animal Care and Use Committee of the University of Texas Medical Branch, Galveston, Texas and conducted according to the National Institutes of Health Guide for the Care and Use of Laboratory Animals (8^th^ edition, National Research Council).

#### *In vitro* transfections

H19-7-IR cell line was purchased from ATCC (American Type Culture Collection). One day before transfection, H19-7 cells were plated in 24 well plates in 500 μl of growth media without antibiotics and at a density of 40,000 cells per well. In a 96 well plate, 20 pM of each siRNA oligo for nNos and GPX 1 were diluted in 50 μl of Opti-MEM I reduced serum medium and mixed by rocking the plate back and forth. In separate wells of the same 96 well plate, 1 μl of Lipofectamine 2000 was diluted into 50 μl of Opti-Mem I reduced serum medium, mixed gently by rocking the plate back and forth, and incubated at room temperature for 5 min. The diluted siRNA oligos were then combined with the diluted Lipofectamine 2000, mixed, and incubated for 20 min at room temperature. The entire complex was added to each well in the 24 well plate that contained H19-7 cells. H19-7 cells transfected with GPx-1 siRNA oligos were incubated at 34°C in 10% CO_2_ for 72 h and then harvested for RNA isolation. H19-7 cells transfected with nNos siRNA oligos were incubated at 34°C in 10% CO_2_ for 96 h and harvested for RNA isolation.

#### RNA isolation

Media was removed and cells were rinsed in 1X PBS. PBS was removed before 500 μl of Ultraspec lysis buffer was added to each well of the transfection and pipetted up and down to aid in cell lysis. Lysate was collected in 1.5 ml tubes and 100 μl of chloroform was added to each sample. Total RNA was extracted by centrifugation at 12,000 g at 4°C. Aqueous phase was transferred into a new tube and RNA was precipitated by adding an equal volume of isopropanol and centrifuged at 12,000 g 4°C. The supernatant was removed before the RNA pellet was washed twice in 75% ethanol and centrifuged at 7500 g at 4°C. The RNA pellet was re-suspended in 20 μl of nuclease free water. Total RNA was DNase treated at 37°C for 20 min to remove any traces of genomic DNA contamination.

#### cDNA synthesis and qPCR

Reverse transcriptase reactions were performed with reagents from the Taqman Reverse Transcriptase Reagents Kit (Applied Biosystem) following manufacturer’s protocols. Real time PCR was performed on a MX3000P Quantitative PCR System (Agilent Technologies). Taqman primer and probe sets for genes nNos, GPx-1, and Gapdh were purchased from (Applied Biosystems), and PCR was performed with Taqman Gene expression master mix, primer sets, and probe sets according to manufacturer’s protocols. Gapdh was used for normalization of expression of nNos and GPx-1. Fold changes were calculated in the Agilent Technologies software program, which compared each transfection group to a negative control.

#### Cloning of AAV siRNA constructs (Vector Biolabs)

All small hairpin RNA oligos were designed as follows: RNAi sequence + "TTCAAGAGA" hairpin loop + anti-sense RNAi + TTTTTT (termination signal) + MluI restriction site (for clone selection). shRNA oligos were synthesized and PAGE purified, and annealed into (BamHI/HindIII) sites of pAAV-U6-eGFP vector. Positive clones were selected using MluI digestion, and prepared with endo-free Mega kit from Qiagen. We co-transfected this pAAV-shRNA plasmid with pAAV1-trans (rep-cap) plasmid and pAd Helper plasmids in 1 cell factory of HEK293 cells for viral packaging. Cells were harvested after 72 hrs, and cell pellets were freeze & thawed 3x before being loaded into the CsCl gradient for overnight centrifuge. AAV fraction were collected and repeated with another round of CsCl gradient. Final AAV stocks were desalted and titered using qPCR.

#### Animals

Adult male Sprague-Dawley rats (350 g–400 g) from vendor Charles Rivers (Portland, Maine) were housed two per cage with food and water ad libitum in a vivarium with these constant conditions: light cycle (6∶00–18∶00) temperature (21°C–23°C), and humidity (40%–50%).

#### Surgical preparation–fluid percussion injury and hippocampal viral injection

Rats were anesthetized with isoflurane in an anesthetic chamber, intubated, and mechanically ventilated with 1.5–2.0% isoflurane in O_2_: room air (70:30) using a volume ventilator (EDCO Scientific, Chapel Hill, NC). Rats were prepared for parasagittal fluid-percussion TBI as previously described [37]. Rats were placed in a stereotaxic head frame and the scalp was sagittally incised. A 4.0 mm diameter hole was trephined into the skull 2.0 mm to the right of the sagittal suture and midway between lambda and bregma. A modified Luerlok syringe hub was placed over the exposed dura, bonded in place with cyanoacrylic adhesive and covered with dental acrylic. Isoflurane was discontinued and rats were connected to the fluid percussion trauma device. They were subjected to severe (2.3 atm) fluid-percussion TBI immediately after the return of a withdrawal reflex to paw pinch. After TBI or sham injury, rats were disconnected from the fluid percussion device and righting reflex was assessed every 60 s until a normal righting reflex was observed. Rats were then placed on 2% isoflurane before wound sites were infused with bupivicaine and sutured with prolene. Isolflurane was discontinued and the rats were extubated and allowed to recover in a warm, humidified incubator. Rats that were to receive an adeno-associated virus were placed back into the stereotaxic head frame and prepared for a hippocampal injection 1 h after injury. The virus was injected using a 26 gauge μl Hamilton syringe into the hippocampal CA1 region- (anterior-posterior -3.6, medial to lateral +2.0, dorsal to ventral -2.6) and the hippocampal CA3 region (anterior-posterior -3.6, medial to lateral +2.0, dorsal to ventral +3.6). After the injection, the needle sat for 2 min. Rats were survived for 15 days and sacrificed. Brains were dissected out, frozen immediately on dry ice, and stored at -80°C until tissue processing.

#### Post-surgical monitoring and method of sacrifice

The animals receive approximately 100mg/kg acetaminophen suppository before emerging from anesthesia. Righting reflex is assessed every 60 seconds until a normal righting reflex was observed. Rats are then placed on 2% isoflurane, wound sites are infused with bupivicaine and sutured with prolene. Isoflurane is discontinued and the rats are extubated and allowed to recover in a warm, humidified incubator. When the animal is fully recovered, it is returned to cage with ad libitum food and water. Animals are monitored for signs of infection, severe neurological injury or discomfort. Signs of discomfort or pain in rodents include persistent dormouse position and unwillingness to move, refusal to eat or drink, vocalizations when handled, posturing, aggressiveness, polyphagia of bedding. Rats exhibiting these symptoms are killed immediately (4% isoflurane in an anesthetic chamber followed by decapitation). When rats are moving around the laboratory cages, usually 24 hours after injury, they are returned to their home cages in the animal facility and observed daily for signs of discomfort using the recommended Quantitative Assessment of Pain Independent Variable Score. Scores are stored in each animal’s computerized record. A total score of 8 or above or an individual score of 3 will result in immediate termination by anesthesia with 4.0% isoflurane in an anesthetic chamber followed by decapitation. 15 days after AAV injection or injury alone animals are sacrificed by decapitation, brains immediately dissected out, frozen on dry ice and stored at -80C until further processing.

#### Sectioning and laser capture microdissection

Pyramidal neurons from the CA1/2 and CA3 regions of the hippocampus that expressed the AAV virus were collected using laser capture microdissection (LCM). LCM was performed using frozen 10 μm coronal sections cut on a Leica 1850 CM cryostat (Lecia, Buffalo Grove, IL) that were mounted on pre-cleaned uncoated glass slides (Fisher Scientific, Pittsburgh, PA). When the hippocampus of the brain was reached, every section was collected and preserved at −20°C until sectioning was complete. Immediately after sectioning, frozen sections were thawed at room temperature for 30 s and fixed for 1 min with 75% ethanol. After fixation, slides were rinsed in RNase-free water (1 min), stained with 1% cresyl violet (1 min), rinsed in RNase-free water (1 min x 3), dehydrated in 95% ethanol (30 s), dehydrated in 100% ethanol (30 s) and xylene (3 min × 2), and air-dried for 10 to 15 min in a hood. All solutions were prepared with RNase-free water and the cresyl violet was sterile filtered just before use.

LCM was performed using a PixCell IIe laser capture microscope with an infrared diode laser (Arcturus Engineering, Mountain View, CA). The pyramidal hippocampal neurons in the Ca1/2 and Ca3 regions infected with the AAV-virus were captured on the thermoplastic film of a CapSure Macro LCM Cap (Arcturus Engineering, Mountain View, CA). The smallest laser spot size (7.5 μm) was used with a power setting of 75–100 mW and pulse duration of 0.45-.85 ms. These last two settings were adjusted, as necessary, for optimum capture of the cells. The Capsure Macro caps containing the hippocampal neurons were transferred to a 0.5 ml tube containing 100 μl of lysis buffer, vortexed, and stored at -80°C until RNA isolation.

#### Total RNA isolation- reverse transcription

Total RNA was isolated using the RNAqueous Micro-Kit (Ambion) following manufacture protocol. Total RNA was DNase treated for 20 min at 37°C. Then, 2ng of total RNA was reverse transcribed using the High Capacity Kit (Thermo-Scientific), according to manufacture protocol.

#### Microarray analysis

Pooled total RNA from hundreds of laser captured hippocampal pyramidal neurons (4.0 ng) from individual rats was sent to GenUs Biosystems for microarray analysis. We prepared three biological replicates for each group (naïve control, TBI, TBI+nNOS siRNA, TBI+GPx-1 siRNA). Total RNA samples were quantitated by UV spectrophotometry (OD260/280). Quality and quantity of Total RNA was assessed using an Agilent Bioanalyzer with the RNA6000 Pico Lab Chip (Agilent Technologies). Labeled cRNA was prepared from total RNA samples. Briefly, the Poly(A)+ RNA population within total RNA was amplified using Arcturus RiboAmp HS reagents (Molecular Devices, Sunnyvale, CA). Alternatively, MessageAMP II (Applied Biosystems, Foster City, CA) was used. After a second round of reverse transcription, second-strand cDNA synthesis, and purification of double-stranded cDNA, *in vitro* transcription was performed using T7 RNA polymerase in the presence of Biotin-11-UTP.

The quantity and quality of the cRNA was assayed by spectrophotometry and on the Agilent Bioanalyzer. Then, 1 μg of purified cRNA was fragmented to uniform size and applied to Agilent Whole Rat Genome microarrays (rat 4X44K arrays, Agilent Technologies, Santa Clara, CA) in hybridization buffer. Agilent Whole Rat Genome microarrays are comprised of approximately 41,000 60-mer probes designed to conserved exons across the transcripts of targeted genes. These probes represent well annotated, full length, and partial human gene sequences from major public databases. Arrays were hybridized at 65°C for 17 h in a rotating incubator and washed at 37° C for 1 min. After staining with Streptavidin-Alexa555, rinsed and dried arrays were scanned with an Agilent G2565 Microarray Scanner (Agilent Technologies, Santa Clara, CA) at 5 μm resolution. Agilent Feature Extraction software was used to process the scanned images from arrays (gridding and feature intensity extraction), and the data generated for each probe on the array was analyzed with GeneSpring GX v7.3.1 software (Agilent Technologies, Santa Clara, CA). To compare individual expression values across arrays, raw intensity data from each gene was normalized to the median intensity of the array. Genes were removed for further analysis if at least one replicate sample was not above background intensity. Further filtering was performed to only include genes whose values were within 50% for biological replicate samples. The filtered gene list was queried for genes that have ratios greater than 2.0 and less than 0.5 (2-fold changes) in TBI +siRNA relative to sham controls. Fold changes were then imported into Ingenuity Pathway Analysis Software.

#### Real-time PCR

Real-time PCR was performed on a MX3000P Quantitative PCR System (Agilent Technologies). Taqman primer and probe sets for genes nNos, GPX1, and GAPDH were purchased (Applied Biosystems), and PCR was performed with Taqman Gene expression master mix for primer and probe sets, according to manufacture protocol. Gapdh was used for normalization of expression of nNos and GPx-11. Fold changes were calculated in the Agilent Technologies software program, which compared experimental groups to Sham controls.

#### Neuronal counting

Five days after injury, the rats were anesthetized before brains were removed, immediately frozen on dry ice, and stored at -80°C. Tissue was frozen in O.C.T. (Tissue Tek) and 25 μM sections through the hippocampus were cut on a cryostat, placed on superfrost plus slides, and fixed for 1 min with 75% ethanol. After fixation, slides were briefly rinsed in RNase-free water (1 min), stained with 1% cresyl violet (1 min), rinsed in RNase-free water (1 min × 3), dehydrated in 95% ethanol (30 s × 2), 100% ethanol (30 s × 2) and xylene (3 min × 2). Sections were stained with Fluoro-Jade (a marker for neuronal cell injury) and dried at room temperature for 1 h. Every fourth section was used for counting Fluoro-Jade positive neurons in the Ca1 and Ca3 regions using Stereo Investigator (Version 9). Estimation of injured neurons was performed using the Optical Fractionator probe. Counting of Fluoro-Jade positive neurons was made at pre-determined intervals (x = 400; y = 400 with a counting frame (x = 400 um; y = 400 um) superimposed on the tissue section image. This procedure sampled 100% of all injured Fluoro-Jade positive neurons. An average number of 50 sections were counted in each treatment group. The total number of injured neurons estimated in each treatment group was compared to the total number of injured neurons estimated in each control group and a TBI alone group.

#### Working memory Morris Water Maze

For assessment of hippocampal-dependent working memory, we used a behavioral test which we recently used to show that working memory deficits persist up to a year following the FPI model used in this study [38]. On PIDs 11–15, rats were tested on the working memory version of the Morris Water Maze (MWM), as described previously [38]. Briefly, the water maze consisted of a 1.8 m diameter tank filled with water to a height of 28 cm. This height is 2 cm higher than the invisible platform, which is 10 cm in diameter and 26 cm in height. Rats received four pairs of trials for five consecutive days. Rats were assigned four starting points (N, S, E or W) and four platform locations (1, 2, 3, or 4) in a balanced order to avoid starting points too close to platforms. For Trial 1: Rats were placed in the tank facing the wall at the assigned location and allowed 120 sec to find the platform. For Trial 2: Rats were immediately placed back in the same starting position and again allowed 120 s to find the platform. After Trial 2, the rats rested for 4 min in a heated enclosure followed by the second pair of trials and repeated until 4 pairs of trials were completed. All rats experienced the same sequence of start points and platform locations, which were randomly selected at the beginning of the experiment.

### Statistical analysis

#### Neuronal counting

FJ positive count data were analyzed using analysis of variance for the completely random design experiment. The sham group was included in the experiment for reference only and not used in the data analysis. The four treatment groups were tested at the 0.05 experimental-wise error rate. Multiple comparisons were conducted for nNos (3) and GPx-1 (2) virus groups vs. scramble virus group and scramble virus group vs. TBI alone group using Fisher’s least significant difference procedure with Bonferroni adjustment for 3 comparisons. Statistical computations were carried out using statistical software, the SAS® system, release 9.1 [39].

#### Neuro-behavioral

Morris Water maze data were analyzed using analysis of variance for a two-factor experiment with repeated measures. The two factors were injury group and time (day and trial). Since time was a combination of day and trial, spatial power option in PROC MIXED, the SAS^®^ system release 9.1 [39], was used for the covariance structure.

#### Real-time PCR gene knockdown

Knockdown data were analyzed using one-way analysis of variance. Main effects and interaction were assessed at the 0.05 level of significance (experiment wise). Multiple comparisons were conducted using Fisher’s least significant difference procedure with Bonferroni adjustment for the number of comparisons.

#### Accession codes for microarray data

The gene expression data reported in this paper have been uploaded to NCBI’s Gene Expression Omnibus (GEO) under the accession number GSE92363.

## Results

### Knockdown of nNOS and GPx-1 *in vitro*

**Fig 1:**
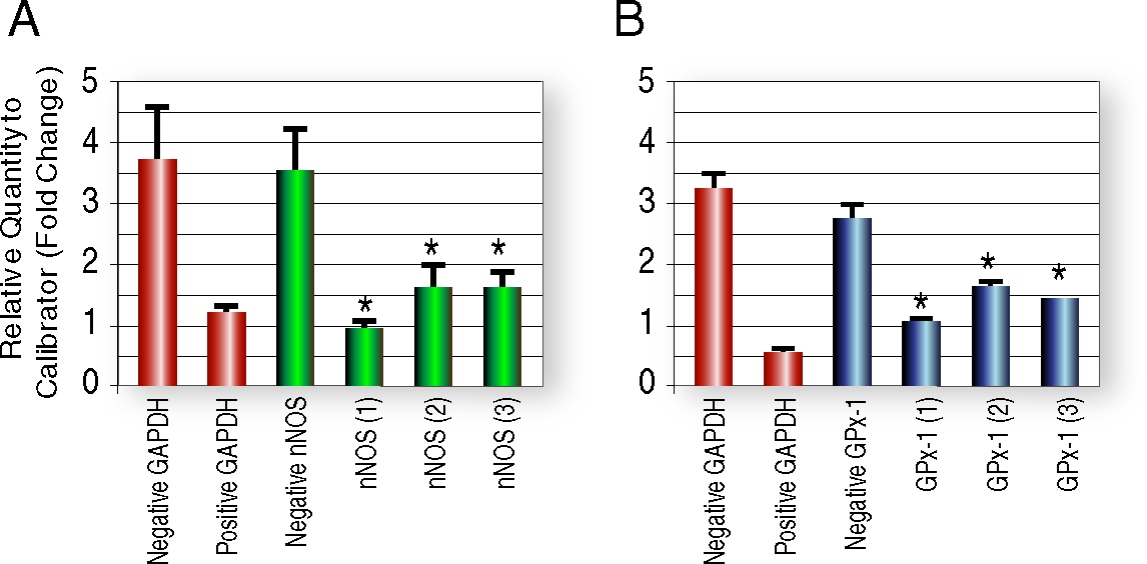
*In vitro* gene knockdown with siRNA oligonucleotides. Knockdown of (A) neuronal nitric oxide synthase (nNOS) and (B) glutathione peroxidase-1 (GPx-1) with *Silencer* pre-designed siRNAs (Ambion) in H19-7 rat hippocampal progenitor cells. All three nNOS and GPx-1 siRNA oligos significantly reduced gene expression in cultured cells.

Our first question was if *in silico* designed siRNAs can effectively reduce expression levels of selected gene targets, nNOS and GPx-1, in a rat hippocampal cell line (H-19). Gene-specific siRNAs can have off-target effects on unrelated genes [40]; therefore, it was critical to show that siRNAs targeted to different regions of the same gene can produce the same effects. We tested three commercially available siRNA oligos targeted to three different regions of nNOS and GPx-1 mRNA (S1 Fig) in H-19 cells (Fig 1A, Fig 1B). For each gene, all three siRNA oligos significantly reduced mRNA levels. These sequences, for all six siRNAs as well as a scrambled siRNA control, were cloned into the AAV 2/1 viral backbone and high titer viruses were produced (Vector Biolabs) for *in vivo* TBI studies.

#### AAV siRNA constructs are expressed in rat hippocampus

Rats were prepared for fluid percussion (FPI) TBI or sham injury, as previously described [37,41], then were assigned into nine groups: 1) sham injury (SHAM); 2) TBI; 3) TBI + AAV scrambled virus (TBI+SV; control); 4–6) TBI + 3 nNOS siRNA constructs; 7–9) TBI + 3 GPx-1 siRNA constructs. Each AAV-GFP labeled siRNA construct was injected using stereotaxic coordinates (see Methods) into the CA1/2 and CA3 sub regions of the hippocampus in two separate injections. All seven AAV constructs, including the scrambled siRNA vector, were expressed in hippocampal sub regions.

**Fig 2:**
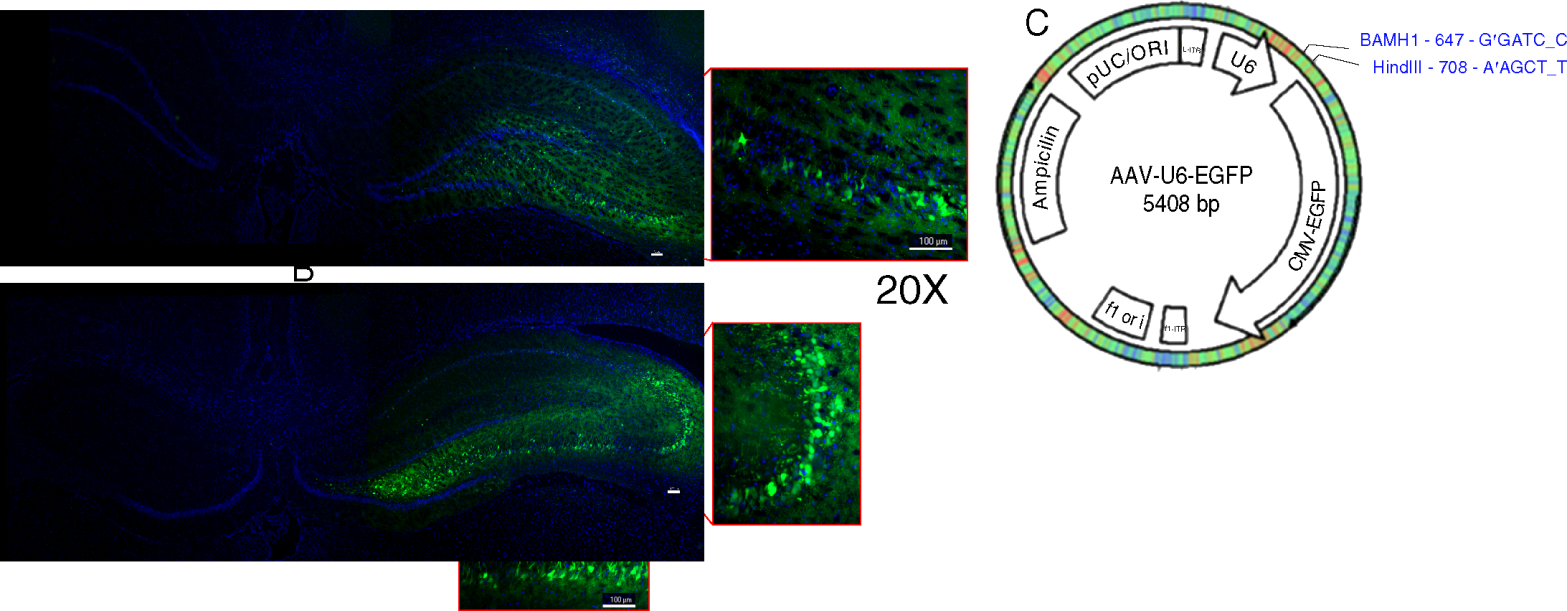
*In vivo* expression of AAV constructs. Specific transduction of rat hippocampal neurons with (A) nNOS (3) and (B) GPx-1 (2) AAV siRNA constructs. (C) All three nNOS and GPx-1 siRNA sequences as well as the scrambled AAV siRNA sequence were cloned into the BamH1/HindIII site of AAV-U6-EFP plasmids. All seven AAV siRNA vectors effectively transduce and are expressed in hippocampal pyramidal neurons.

Please note that after performing the behavioral studies with all seven viral constructs in the Morris Water Maze (see below) and analysis of qPCR data, we selected the siRNA construct for each gene that in TBI rats showed the most promising trends towards supporting our hypothesis that knocking down nNos [nNOS (3)] would improve and that knocking down GPx-1 [GPx-1 (2)] would worsen neurobehavioral outcome. If an AAV construct that knocked down gene expression did not show promise in the behavioral tests, we reasoned that downstream analyses, i.e. microarray analysis may be moot. The nNOS (3) and GPx-1 (2) siRNA AAV constructs were subsequently used for stereological and microarray analysis in the hippocampus two weeks post-sham or FPI injury. Two weeks after stereotaxic injection, we found that the majority of pyramidal neurons in the CA1-CA3 subfields were infected with recombinant viruses (Fig 2). Injection of AAV can elicit an immune response, causing cytopathic brain damage that confounds results of gene silencing. Therefore, to assess the magnitude and extent of possible virus-induced inflammation, we performed immunohistochemical analysis of CD68 and TCR immunoreactivity in brain sections from rats treated with SHAM, TBI, TBI+ SV, and all three TBI+ nNOS or TBI+ Gpx-1AAV vectors. No discernable increase in immunostaining, beyond that induced by TBI, was detectable in any AAV treated brains (S1 Fig and S2 Fig).

#### LCM and qPCR analysis of nNOS and GPx-1 expression *in vivo*

**Fig 3:**
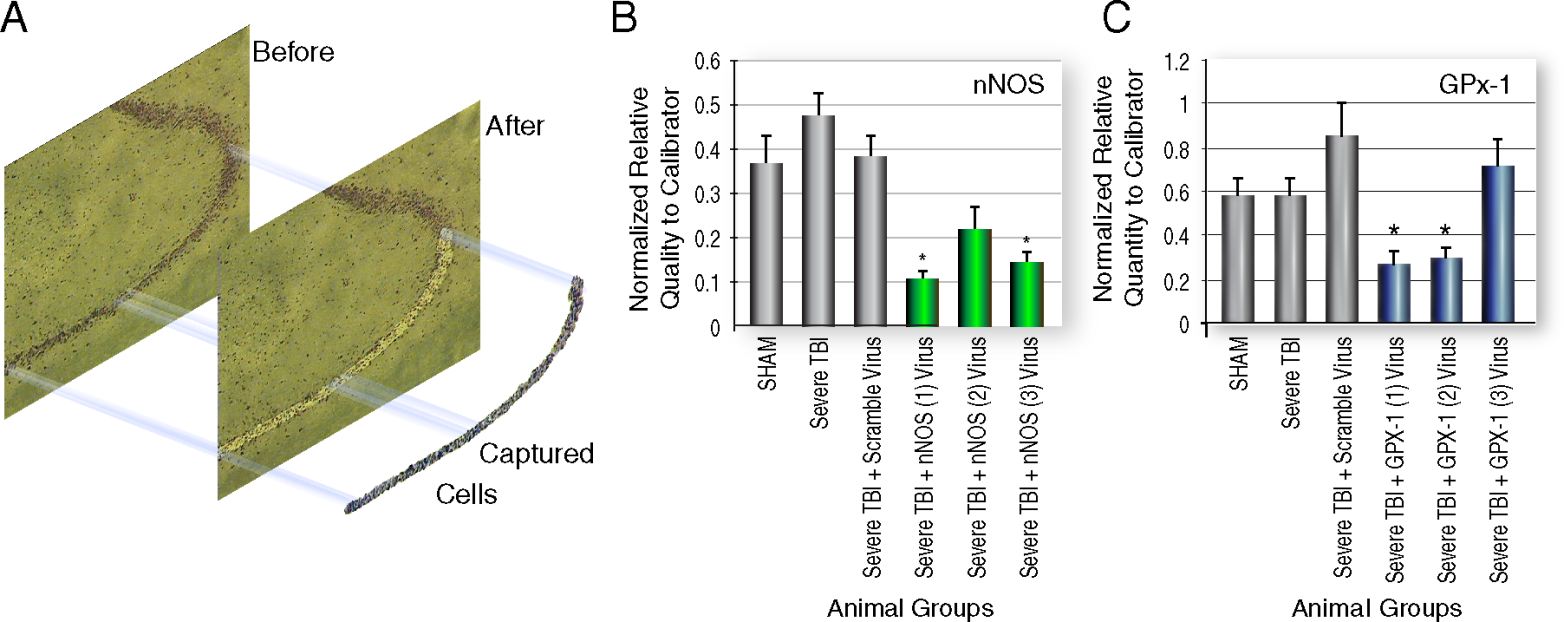
Quantitative real-time PCR analysis of laser captured hippocampal neurons. (A) Laser capture microdissection of AAV siRNA treated hippocampal pyramidal neurons for subsequent quantitative real time PCR (qPCR) analysis. Nissl stained image showing the precise capture of pyramidal neurons for subsequent qPCR analysis of nNOS or GPx-1 expression. (B, C) QPCR analysis of nNOS and GPx-1 expression in laser captured neurons from brains of all control, TBI and virus-treated rats two weeks after siRNA virus treatment.

We assessed the efficiency of gene knockdown *in vivo* with all three viral vectors by qPCR analysis of laser captured pyramidal neurons 15 days after injury/sham surgery. Because the LCM staining protocol washed out GFP fluorescence, we viewed frozen sections not subjected to ethanol fixation and dehydration protocols of LCM. These sections clearly showed GFP fluorescence associated with AAV transduction in hippocampal neurons. We then laser captured neurons from the same regions in immediately adjacent sections. The clean laser capture of hippocampal cells—the region of the hippocampus captured on macrocaps–is seen in nissl-stained coronal sections (Fig 3A).

QPCR analysis showed the three control and injury groups, Sham, TBI and TBI+SV, were not significantly different from each other for NOS or GPx-1 expression. This result was expected since expression levels of most TBI-induced genes are likely to have normalized by 15 days post-injury. Two of the three GPx-1 siRNA vectors, GPx-1 (1) and GPx-1(2), were significantly knocked down *in vivo* (0.27 and 0.30 vs. 0.85; *P* = 0.0005 and 0.001, respectively; Fig 3B). All three nNOS siRNA vectors reduced gene expression, and both TBI + nNOS (1) and TBI + nNOS (3) were significantly lower than TBI+SV (0.11 vs. 0.38, respectively, *P* = 0.003). Although the TBI+NOS (2) group was lower than TBI+SV, this result did not reach statistical significance (0.19 vs. 0.38; *P* = 0.07; Fig 3C). Overall, the siRNA AAV vectors effectively reduced expression of targeted genes *in vivo*.

#### Stereological counting of degenerating neurons after AAV siRNA treatment

**Fig 4:**
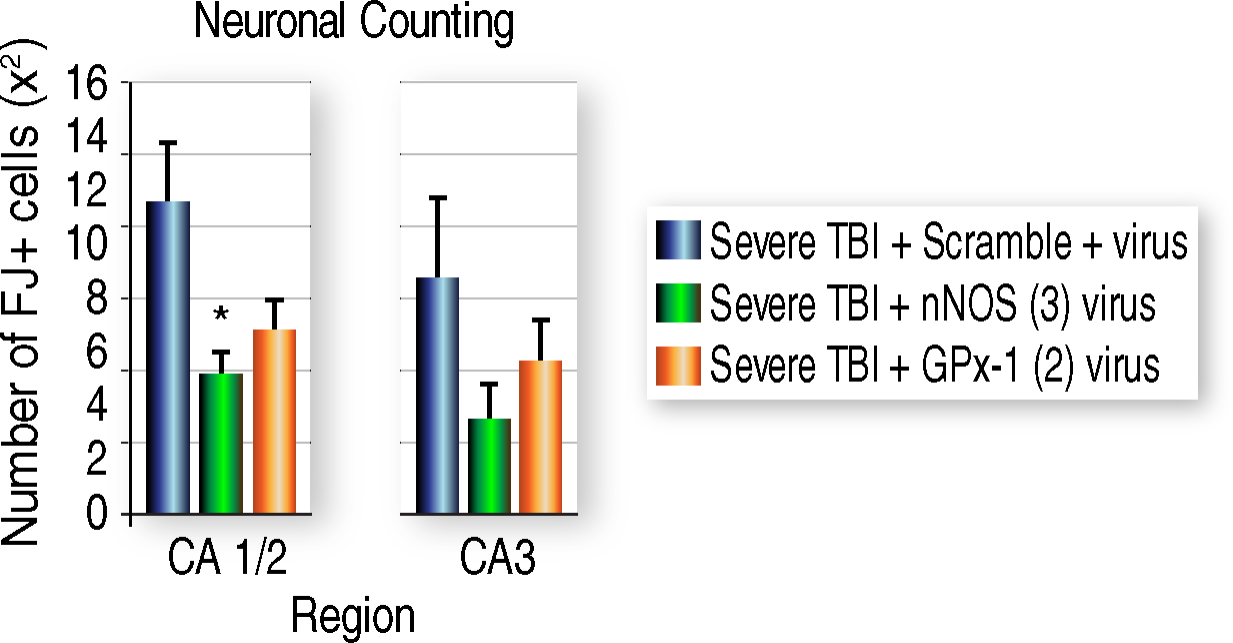
Stereological assessment of neuronal injury. GPx-1 (2) and nNOS (3) were selected for *in vivo* assessment of neurodegeneration based on initial results of the qPCR and neurobehavioral tests. Only AAV nNOS (3) significantly reduced numbers of degenerating, Fluoro-Jade-positive (FJ+) cells in the TBI rat hippocampus.

We hypothesized that knocking down nNOS will reduce hippocampal degeneration because of its association with brain pathology. Conversely, given its potentially protective role as an antioxidant, we hypothesized that GPx-1 knockdown will exacerbated neuronal injury. To determine if silencing nNOS or GPx-1 reduces or increases neurodegeneration in TBI rats, we stained frozen rat brain sections with Fluoro-Jade (FJ), a marker of neurodegeneration [42], and determined numbers of dying (FJ+) neurons using the Optical Fractionator probe and Stereo investigator software. *In vivo* delivery of AAV-nNOS (3) siRNA reduced numbers of dying, degenerating hippocampal neurons, but the GPx-1 (2) vector had no discernable effects on TBI-induced neurodegeneration (Fig 4).

#### AAV siRNA effects on hippocampal-dependent learning and memory

**Fig 5:**
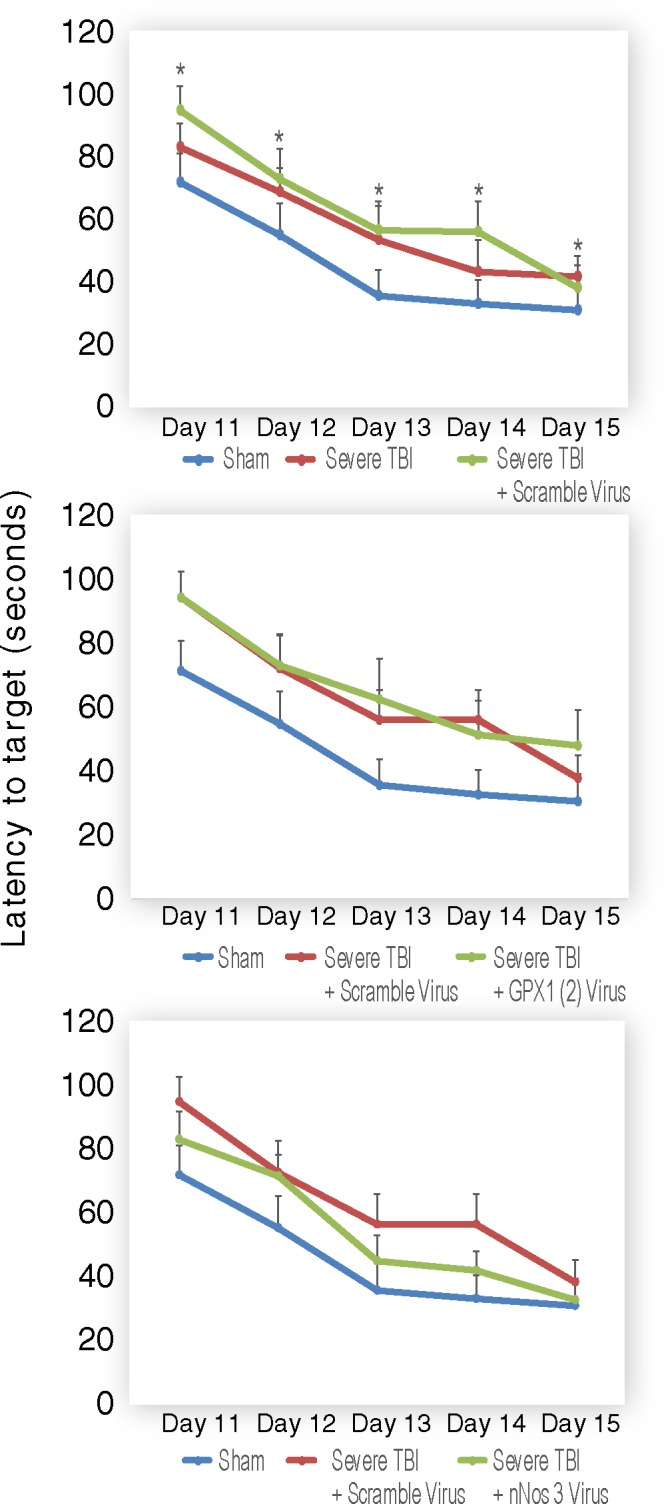
Neurobehavioral assessment after AAV siRNA treatment.

We compared the effects of virus treatments on hippocampal-dependent working memory using the Morris Water Maze (MWM) test. Cell injury induced by viral injection alone can cause additional off-target effects; therefore, MWM results of nNOS or GPx-1 siRNA treated TBI rats were compared to TBI rats with scrambled AAV siRNA. TBI rats with scrambled siRNA AAV treatment performed significantly worse than sham injured rats. To simplify, only the results of Trial 2 are shown in Fig 5. While TBI was not significantly different from SHAM (*P* = 0.10) or TBI+SV (*P* = 0.23), TBI+SV was significantly different from SHAM (*P* = 0.01) (Fig 5 *upper*). Comparison of TBI + GPx-1(1), TBI+GPx-1(2), and TBI+GPx-1(3) to TBI + SV revealed no statistically significant differences (S4 Fig). Nevertheless, TBI+GPx-1(2) showed a trend towards longer latencies than TBI+SV over both trials and days, suggesting a potentially harmful influence (Fig 5 *middle*). Comparison of TBI+ nNOS(1), TBI+nNOS(2), TBI+nNOS(3) to TBI+SV revealed no statistically significant differences (S4 Fig). However, the latency of TBI+nNOS(3) was observationally shorter than TBI+SV (Fig 5 *lower*), suggesting a possible protective effect of nNOS(3) siRNA.

The TBI alone group and the TBI plus scrambled AAV (SV) siRNA group showed significantly worse latencies then the sham alone group (p<0.05). The TBI alone group and TBI plus SV group showed no differences within the groups; thus TBI plus SV was used as the control group for the nNos and GPX-1 constructs. Even though overall swim times across trials and days of all 3 TBI+nNOS groups were observed to be shorter than TBI+SV group, this was not statistically significant. All 3 of the GPX-1 constructs were not significantly different from TBI plus SV (Data for all constructs shown in S3 Fig).

#### Gene expression profiles reflect gene-specific knockdown

**Fig 6:**
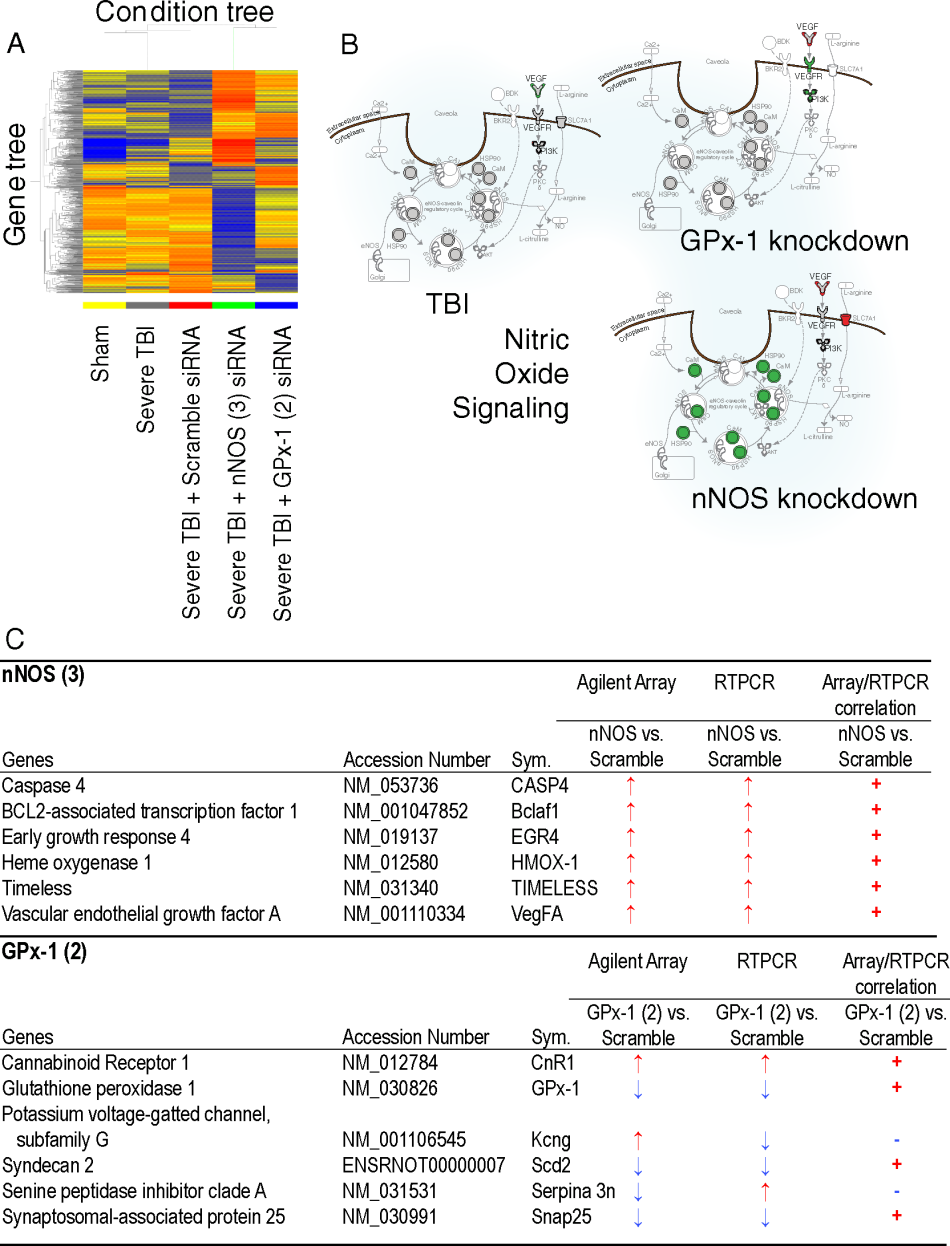
Gene expression analysis of AAV treated rat brains. (A) Heat map from microarray analysis (rat Agilent whole genome arrays) of hippocampal gene expression two weeks after siRNA virus treatment. NNOS knockdown has strong effects on injury-associated gene expression. The effects of GPx-1 knockdown were more subtle. (B) AAV mediated gene silencing was very specific; only nNOS knockdown had effects on the canonical nitric oxide signaling pathway. (C) Confirmation of virus induced changes in genes affected by AAV siRNA treatments. All selected gene targets of nNOS knockdown were confirmed and four of six gene targets of GPx-1 knockdown showed expression trends that matched the microarray results.

Knocking down disease-causing genes affects brain function [43–46], but the consequences on downstream genes and pathways that regulate cognitive function and behavior have not been fully explored. Since the outcomes of knocking down or silencing nNOS or GPx-1 in hippocampal neurons have not been described at the genomic level, we leveraged our ability to laser capture pure pools of virus-infected neurons to determine the genomic effects of siRNA in hippocampal pyramidal neurons. To delineate gene expression patterns altered by AAV-mediated siRNA treatment, we isolated total RNA from laser captured hippocampal pyramidal neurons and performed microarray analysis using Agilent rat whole genome arrays (Gene Expression Omnibus accession # GSE92363). Hierarchical clustering shows that nNOS or GPx-1 knockdown profiles are distinct from TBI or sham injury alone. AAV scrambled virus did not significantly alter the TBI-induced gene expression profiles (Fig 6). Conversely, nNOS or GPx-1 knockdown had consistent effects on nitric oxide and GPx-1 regulated genes. We surmised the effects of nNOS and GPx-1 siRNAs were gene specific by studying the impact of gene knockdown on canonical nitric oxide signaling (Fig 6B, shown enlarged in S5 Fig); it is clear only nNOS regulated genes were altered by nNOS siRNA. To validate microarray expression of siRNA treated cells, we obtained total RNA from laser captured hippocampal neurons infected with AAV vectors (TBI rats treated with scrambled, nNOS, or GPx-1 siRNA) and performed real time qPCR analysis of several genes whose expression was significantly altered after nNOS or GPx-1 knockdown (Fig 6C). We confirmed altered expression of all nNOS affected gene targets and four of six GPx-1 gene targets.

#### Pathways differentially affected by nNOS or GPx-1 knockdown

**Fig 7:**
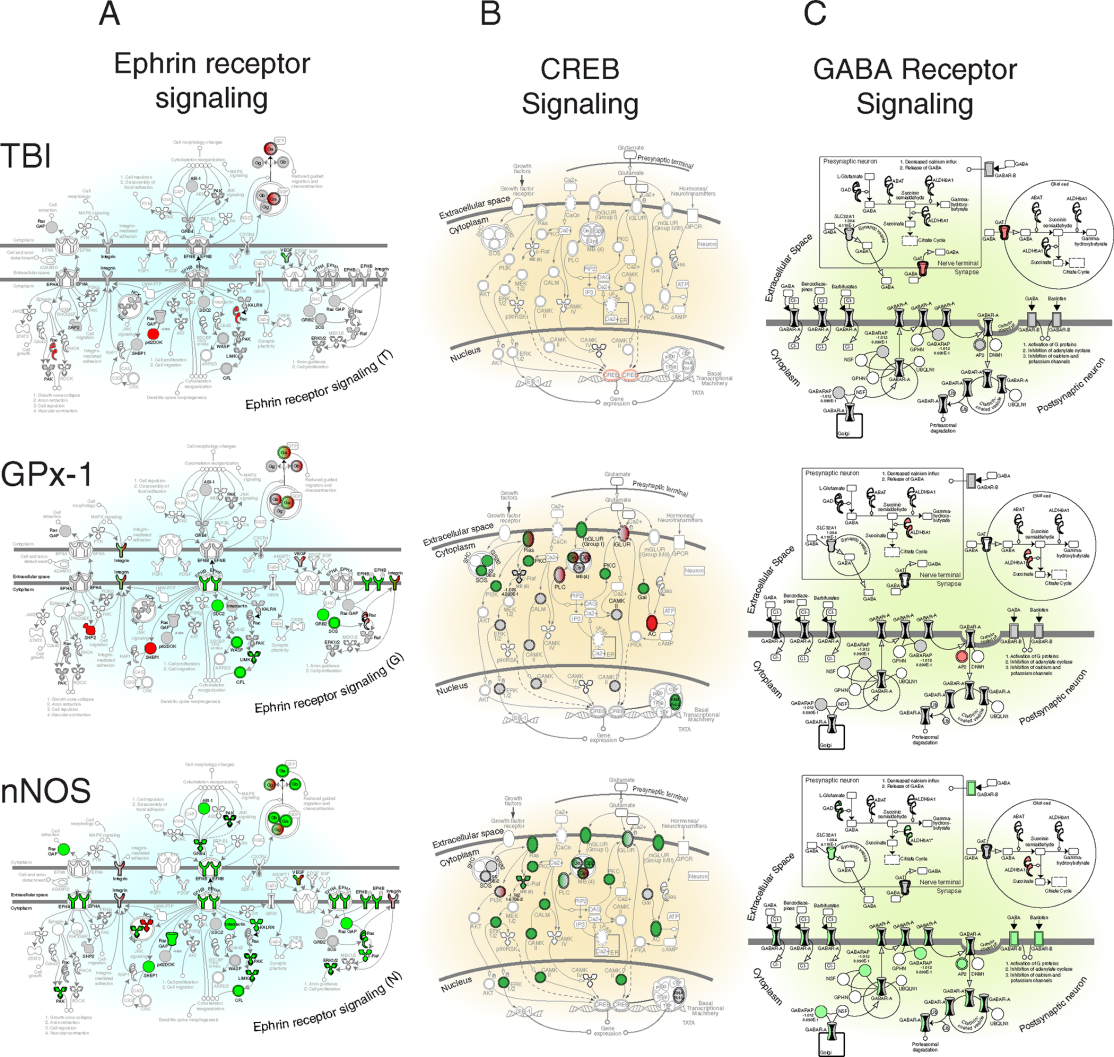
Comparison of AAV-mediated gene silencing on three canonical downstream cell signaling pathways essential for neuronal function. (A) Ephrin receptor signaling (B) CREB signaling (C) GABA receptor signaling.

**Fig 8:**
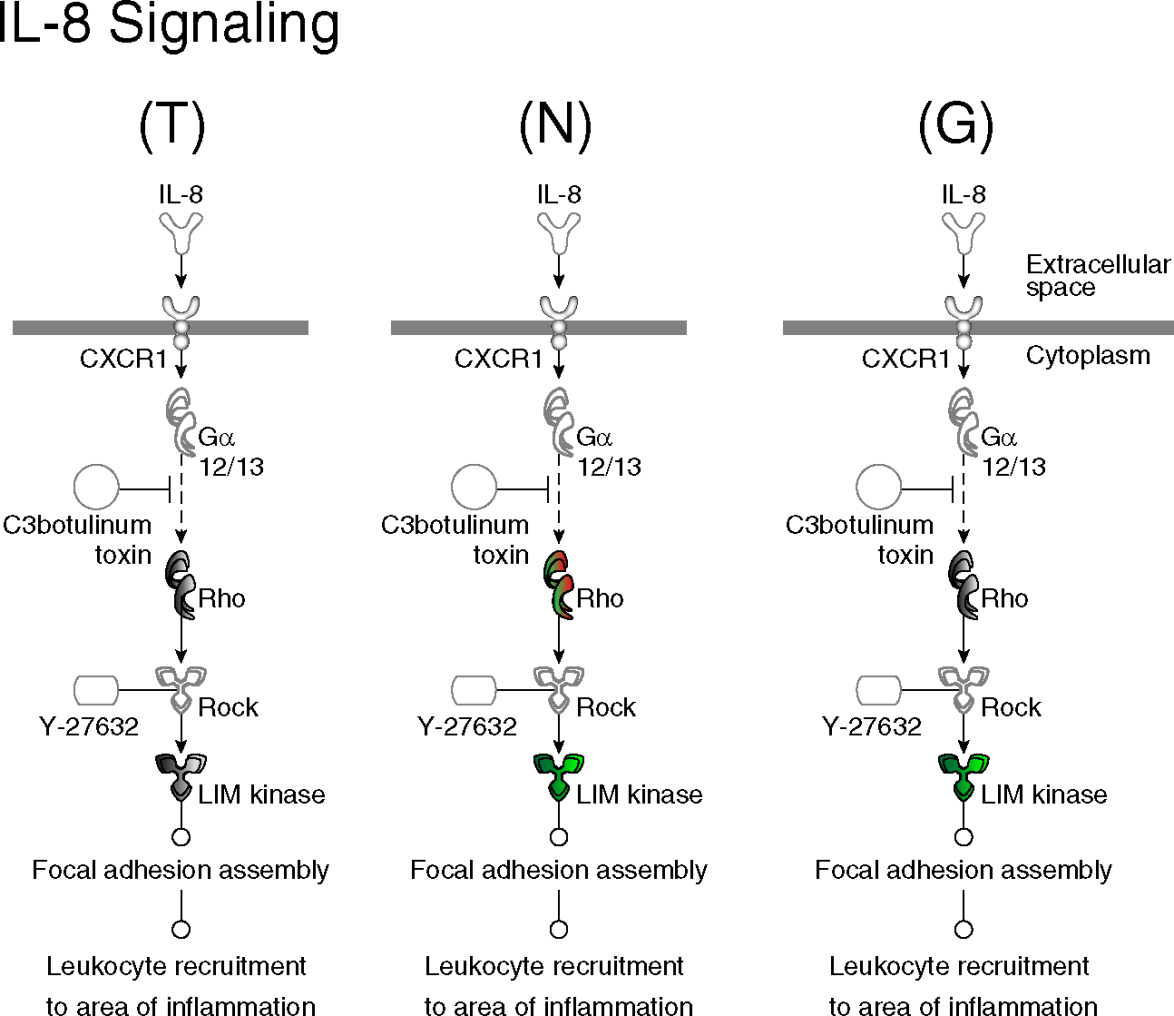
Inhibition of Rock and Lim kinase in Rho-Rock-Lim kinase pathway by AAV-nNOS siRNA suggests a mechanism for AAV nNOS-mediated neuroprotection. (T) = Traumatic brain injury, (N) = nNos (neuronal nitric oxide synthase), (G) = GPx-1 (Glutathione peroxidase-1). Within the pathways, genes that are labeled green are down-regulated and genes that are in red are considered up-regulated compared to uninjured control expression.

The differential effects of AAV siRNA mediated knockdown are best appreciated by looking at the effects on canonical cell signaling pathways regulated by these genes (Fig 7A, Fig 7B, Fig 7C, shown enlarged in S6 Fig, S7 Fig and S8 Fig). We used Ingenuity Pathway Analysis to study effects of gene knockdown in AAV expressing hippocampal neurons. In contrast to the modest effects of gene knockdown on functional outcome measures, both siRNA vectors, but particularly AAV-nNOS siRNA, had a global suppressive effect on several downstream cell signaling pathways. Notably, there was an effect on three signaling pathways involved in neuronal survival, growth, and function (Ephrin receptor, CREB, and GABA receptor signaling) [47]. All three pathways are essential for synaptic function and/or neural plasticity, and GPx-1 gene knockdown downregulated downstream genes in the Ephrin receptor and CREB pathways, as expected; however, inhibition of multiple genes in all three pathways by nNOS siRNA was an unexpected finding. Despite superficial similarities, gene specific effects could be discerned by examination of biologically relevant signaling pathways, i.e. AAV-nNOS siRNA specifically inhibited both Rock and Lim kinase in the Rho-Rock-Lim kinase pathway (Fig 8).

## Discussion

Therapeutically altering gene expression in the brain via gene silencing or gene delivery is one of the great challenges in translational medicine today and yet remains one of the most promising [4]. A primary goal in TBI research is development of therapies that either inhibit expression of deleterious injury-induced signals or enhance endogenous pro-survival signals. Although RNAi has been reported in studies of ischemic brain injury [43], in mice subjected to FPI [48], and *in vitro* stretch models of brain injury [49,50], this study is the first to report the consequences of *in vivo* RNAi in a rat TBI model. Given that AAV is widely used in human clinical trials of gene therapy because of its proven safety profile and efficacy in delivery of genes to diverse cell types [22], this viral vector was the most promising choice for these proof-of-principle studies in our FPI model.

The initial promise of RNAi has been tempered by years of conflicting and disappointing results, which highlight the pitfalls of this gene silencing technique [51]. Thus, this method has not continued to be supported as a powerful interventional tool to induce loss-of-function in a disease-causing gene and to reverse disease pathology for improved functional outcome. The key challenge is in demonstrating causality and showing that knockdown of injury-induced genes causes observable and beneficial phenotypes. Our results suggest single gene knockdown can alter cell fate, i.e. reduce neuronal death, but despite having striking effects on downstream genes, it does not result in significant and visible behavioral changes. In a recent cross-validation study, the authors found an 18.5% failure rate in RNAi gene silencing efficiency across 429 independent studies [52]. This variability could have contributed to confounding results in our studies. Although there are promising clinical trials using RNAi to treat diseases, such as HIV [53], RNAi directed towards a single gene target is unlikely to have desired therapeutic effects, especially in brain injury due to the heterogeneous TBI patient population and complex injury profiles [54]. Despite specific targeting, gene delivery by siRNA vectors is highly complex and there are issues in overcoming critical thresholds of protein knockdown and in effective transduction of targeted cell types; these limitations hinder effective gene silencing in brain disorders [55].

Since the effects of delivering naked siRNAs are transient, using AAV vectors ensures a stable form of siRNA that persists for weeks to months. In this study, we used AAV serotype 2/1, which has an established safety record- AAV has been used in many clinical trials for the past decade- and past research has demonstrated that it is efficient in infecting human neuronal cells [22,56,57]. Although in our study we only measured gene knockdown at 2 weeks after injection, previous studies have shown that AAV persists in the CNS for several months to years [22]. Recent successful gene therapy studies using a modified AAV vector to restore auditory and vestibular function in a mouse model of hearing loss [58] show the translatable value of AAV vectors in safe, long lasting gene therapy for human disease.

We undertook this project with full awareness that deciphering both the positive and negative consequences of gene silencing in injured brain regions would be challenging because of the extreme heterogeneity and complexity of the mammalian brain [59]. Indeed, our confounding results reflect difficulties posed by attempts to modulate expression of single genes in an organ that expresses more genes than any other tissue.

AAV vectors have been used to overexpress pro-survival genes, such as the BDNF receptor trkB in TBI mice; however investigators of this previous study showed that there were no effects on neuronal loss nor any amelioration of TBI-induced cognitive deficits [60]. On the other hand, a recent study reported that pharmacological inhibition of nNOS in ischemic brain injury showed protective effects [61]. Thus, we hypothesized that AAV-mediated silencing of nNOS, whose increased expression is causally linked to neurodegeneration [62,63], would reduce neuronal death and improve hippocampal-dependent memory. Knocking down nNOS did significantly reduce neurodegeneration in the injured hippocampus, supporting the postulated pathological role of TBI-induced increases in nNOS expression. Since the goal of this study was to manipulate genes to reduce brain damage and to potentially restore normal function, our results suggest nNOS may still be a therapeutic target in future preclinical studies of TBI. In contrast, the functional effects for GPx-1 were modest. There were no changes in neuronal injury and the potential effects on working memory and other behavioral deficits require further investigation.

Although we performed behavioral analysis of working memory in siRNA treated rats, resource constraints limited us in expanding our analysis to include other brain functions, such as the effects on circadian rhythms. One original motivation for selecting nNOS as an RNAi target was its involvement in regulating the sleep-wake cycle [64]. Sleep-wake cycles are disturbed in TBI patients [65] and we have shown that circadian rhythm genes are dysregulated in rat brains after TBI [66]. A recent study implicated nNOS in degeneration of orexin/hypocretin neurons that are essential components of regulation of sleep-wake cycles [67]. Thus, in future studies, sleep-wake rhythms could be evaluated in brain-injured rats after knock-down of nNOS expression in orexin/hypocretin neurons.

Although Ingenuity Pathway analysis gave confounding results, it helped to shed light on one mechanism for how inhibition of nNOS signaling reduces cell death. The finding that genes in rho-rock-lim kinase signaling were specifically downregulated in nNOS siRNA treated rat brains are concordant with a recent study that showed inhibitors of Rock2-Lim pathway reduce neurodegeneration [68]. The remarkable specificity of siRNA treatment is reflected in the lack of effects on the nitric oxide signaling pathway in TBI or GPx-1 siRNA rats; only nNOS downstream genes were affected by nNOS siRNA. The sheer number of altered genes in our microarray analysis suggested both nNOS and GPx-1 may play regulatory roles in diverse cell signaling pathways. Contrary to our original hypothesis, the paradoxical down-regulation of genes in known pro-survival and pro-growth pathways, such as Creb and ephrin receptor signaling, implicates involvement of other unknown pro-survival genes in the reduction of neurodegeneration after AAV-nNOS siRNA treatment. It is difficult to reconcile how suppression of pro-survival pathways, such as CREB, could improve neuronal survival since knockdown of CREB increases apoptosis [69]. These confounding data indicate functional outcomes cannot be easily predicted or inferred based on genomic changes.

Despite significant alterations in downstream genes with each AAV siRNA vector, we could not discern any observable changes in hippocampal-dependent memory tests. We speculate that the knockdown of nNOS expression in the hippocampus was insufficient to significantly improve working memory deficits because multiple brain regions subserve working memory. For example, in studies of working memory in humans and nonhuman primates, the frontal cortex is important [70] and functional connectivity studies show memory is distributed over many brain regions [71]. Therefore, our original hypothesis needs to be modified to allow for involvement of other regions, such as the thalamus, that are essential for synchronous activity of the prefrontal cortex and hippocampus during working memory tasks [72]. We also cannot rule out that surviving hippocampal neurons after nNOS knockdown may not play a role in working memory circuits. In mice, knockdown of Hes1, a negative regulator of neurogenesis, improved cognitive function in the MWM after TBI [73] which suggested that RNAi could improve memory in the right context. Indeed, after we had performed our original experimental design, a study of nNOS knockout mice suggested that working memory deficits in these nNOS KO mice were due, in part, to deletion of nNOS expression in the PFC during cortical development; nNOS deletion impaired its binding to another gene disrupted-in-schizophrenia 1 (DISC1), which is also important for cognitive function [74].

The lack of significant differences in a hippocampal-dependent working memory task, MWM, after AAV siRNA treatments does not refute the importance of the hippocampus in working memory and other hippocampal-dependent cognitive functions. Recent studies in bats confirmed the role of the hippocampus in goal-directed spatial navigation [75]. Effects of gene knockdown on downstream cell signaling pathways could have unpredictable changes on the functional outcome measures we employ to assess effects of gene silencing. While we did not detect any effects of the AAV siRNA vectors on hippocampal-dependent behavioral tests, other studies have found a single injection of AAV alters endogenous gene expression a year post-injection [76]. Thus, our study does not preclude long term genomic changes that may result from acute AAV siRNA-mediated reduction in degenerating neurons in the hippocampus; these changes may preserve other cognitive functions not tested in our study.

As a limitation, AAV treated rats were tested with only one hippocampal function test at one time point. We acknowledge our focus on optimizing siRNA *in vivo* expression and on characterizing molecular changes rather than behavioral phenotypes. Other tests might detect significant changes in hippocampal-dependent behavior. Nevertheless, the lack of a clear and discernable behavioral phenotype suggests that knocking down any one gene may not alter behavior dependent on coordinated functions of hundreds of genes in an equally large number of neuronal circuits. It is also possible the lack of a discernable behavioral phenotype is due to activation of endogenous cell signaling processes that oppose the effects of knocking down nNOS or GPx-1. Combining RNA therapeutics with other treatment modalities may be necessary to produce the desired outcomes.

Our study results suggest we might need to manipulate multiple epigenetic or transcriptional regulators to affect major change in behavioral outcome. With the advent of innovative CRISPR-Cas9 techniques [77], we now have additional powerful genomic engineering tools for manipulation of gene expression on multiple gene targets, although this technology, like RNAi, also suffers from off-target effects. On a cautionary note, we have evidence from these CRISPR studies that older gene function studies may not hold up [78]. Thus, any phenotype we describe will need additional validation in other studies. Due to the complexity of the mammalian brain, many problems with specificity of these gene altering techniques must to be worked out before gene silencing becomes disease-modifying.

## Supporting information

S1 Fig Sequences of silencer pre-designed siRNA oligonucleotides. (PDF)

S2 Fig Immunohistochemical analysis of CD68 immunoreactivity in experimental rat brains. (PDF)

S3 Fig Immunohistochemical analysis of TCR immunoreactivity in experimental rat brains. (PDF)

S4 Fig Morris Water Maze, working memory paradigm two weeks post-surgery. (PDF)

S5 Fig Enlargement of nitric oxide signaling (Fig 6B). (PDF)

S6 Fig Enlargement of Ephrin receptor signaling (Fig 7A). (PDF)

S7 Fig Enlargement of CREB signaling (Fig 7B). (PDF)

S8 Fig Enlargement of GABA signaling (Fig 7C). (PDF)
