# Supplementary material for: Effects of AAV-mediated knockdown of nNOS and GPx-1 gene expression in rat hippocampus after traumatic brain injury
Source: PLoS One. 2017 Oct 10;12(10):e0185943. doi: 10.1371/journal.pone.0185943 (PMC5634593; doi:10.1371/journal.pone.0185943)
Supplement: S1 Fig — (PDF) [file pone.0185943.s001.pdf]

S1 Figure.
siRNA sequences for nNOS and GPx-1

Silencer Pre-designed siRNA purchased from Ambion.

Target gene: glyceraldehyde-3-phosphate dehydrogenase
Sense Strand Sequence: GGUCAUCCAUGACAACUUUtt
Antisense Strand Sequence: AAAGUUGUCAUGGAUGACCtt

Target gene: nitric oxide synthase 1, neuronal (siRNA ID 54664)
Sense Strand Sequence: GGUCAAGAACUGGGAGACGtt
Antisense Strand Sequence: CGUCUCCCAGUUCUUGACCtt

Target gene: nitric oxide synthase 1, neuronal (siRNA ID 54760)
Sense Strand Sequence: GGUCUUUCCAAUGUUCACAtt
Antisense Strand Sequence: UGUGAACAUUGGAAAGACCtt

Target gene: nitric oxide synthase 1, neuronal (siRNA ID 54850)
Sense Strand Sequence: GGGCUUCACUACACAUCUGtt
Antisense Strand Sequence: CAGAUGUGUAGUGAAGCCCtc

Target gene: glutathione peroxidase 1 (siRNA ID 52839)
Sense Strand Sequence: GGUGCUGCUCAUUGAGAAUtt
Antisense Strand Sequence: AUUCUCAAUGAGCAGCACCtt

Target gene: glutathione peroxidase 1 (siRNA ID 52931)
Sense Strand Sequence: GGAGGAUGGCAAGAAUGAAtt
Antisense Strand Sequence: UUCAUUCUUGCCAUUCUCCtg

Target gene: glutathione peroxidase 1 (siRNA ID 53015)
Sense Strand Sequence: GGUGAAUGGUGAGAAGGCUtt
Antisense Strand Sequence: AGCCUUCUCACCAUUCACCtc

S1 Figure. Sequences of Silencer Pre-designed siRNA oligonucleotides for glyceraldehyde-3-phosphate dehydrogenase and neuronal nitric oxide synthase (nNOS) and glutathione peroxidase-1 (GPx-1). Each of the three nNOS and three Gpx-1 siRNA oligos target three different regions of the mRNA sequence.
