# Supplementary material for: Effects of AAV-mediated knockdown of nNOS and GPx-1 gene expression in rat hippocampus after traumatic brain injury
Source: PLoS One. 2017 Oct 10;12(10):e0185943. doi: 10.1371/journal.pone.0185943 (PMC5634593; doi:10.1371/journal.pone.0185943)
Supplement: S3 Fig — (PDF) [file pone.0185943.s003.pdf]

S3 Figure.
CD 68 and TCR immunos

TCR alpha/beta (T-cells)

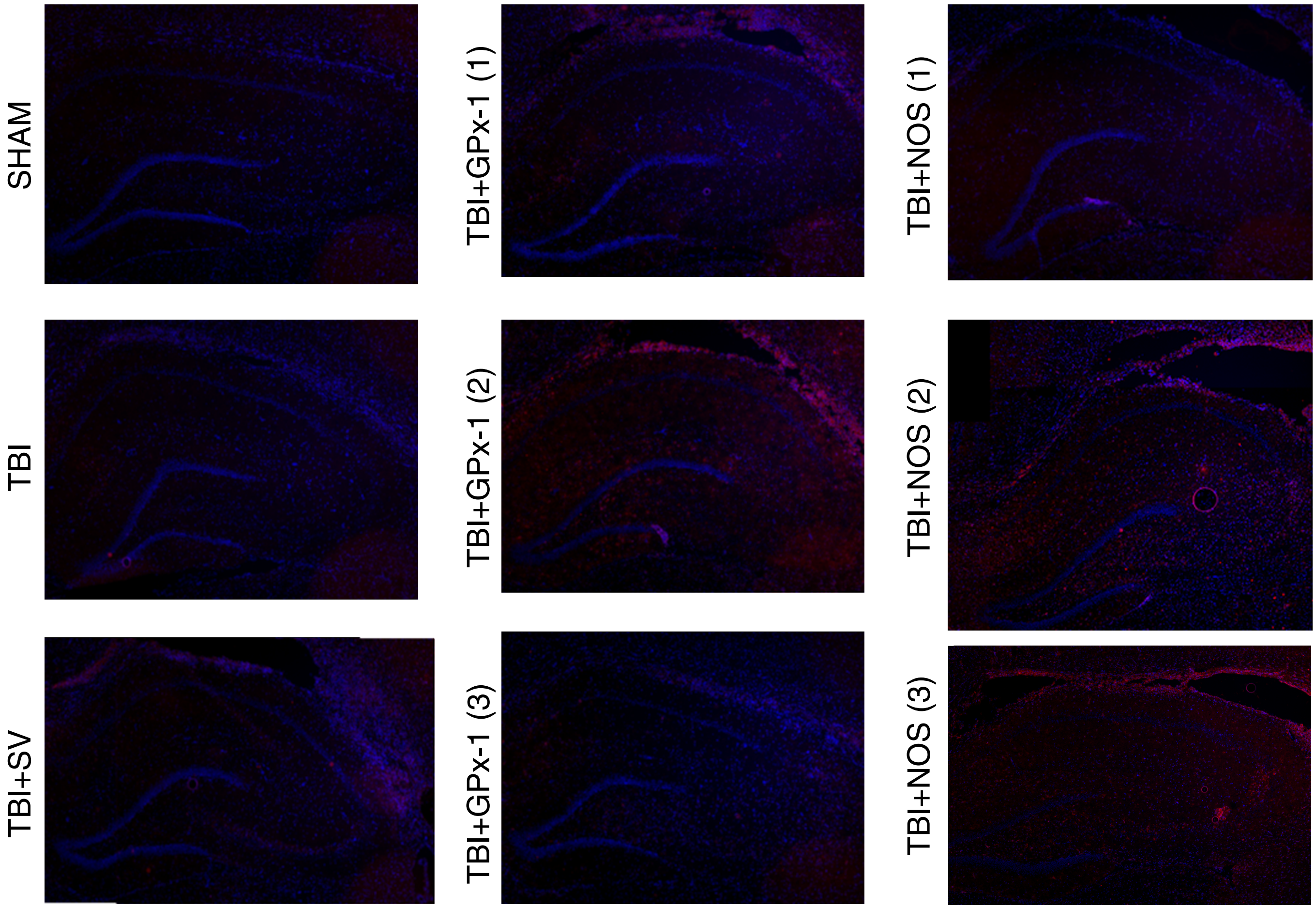

S3 Figure. Immunohistochemical analysis of TCR immunoreactivity in brain sections from rats treated with SHAM, TBI, TBI+ SV, and all three TBI+ nNOS or TBI+ Gpx-1AAV vectors. No discernable increase in immunostaining, beyond that induced by TBI, was detectable in any AAV treated brains suggesting that virus-induced inflammation did not confound the effects of the siRNA viral vectors.
