# Supplementary material for: Effects of AAV-mediated knockdown of nNOS and GPx-1 gene expression in rat hippocampus after traumatic brain injury
Source: PLoS One. 2017 Oct 10;12(10):e0185943. doi: 10.1371/journal.pone.0185943 (PMC5634593; doi:10.1371/journal.pone.0185943)
Supplement: S4 Fig — (PDF) [file pone.0185943.s004.pdf]

S4 Figure.
Working memory water maze 2 weeks post-surgery

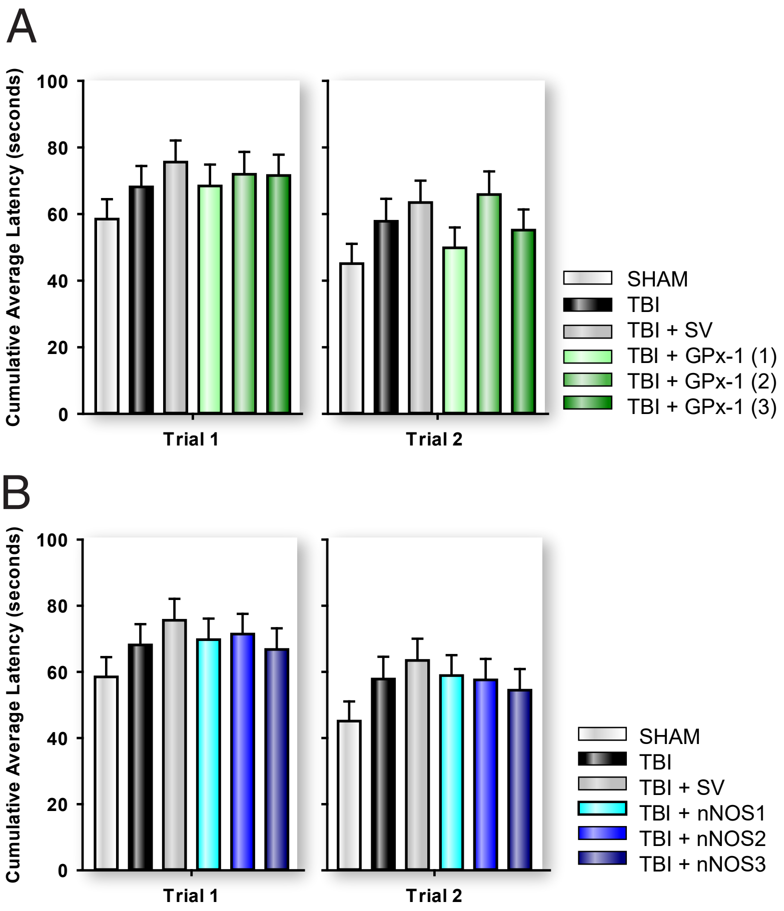

S4 Figure. Morris Water Maze, working memory paradigm two weeks post-surgery. Cumulative average latencies in both trial 1 and trial 2 for rats treated with each of the three nNOS or GPx-1 siRNA constructs are shown compared to latencies from sham control, TBI alone and TBI plus scrambled virus rats. TBI + scrambled virus (SV) treated rats were significantly different from sham rats. All other comparisons were not significant.
