# Supplementary figures and images for: Effects of AAV-mediated knockdown of nNOS and GPx-1 gene expression in rat hippocampus after traumatic brain injury
Source: PLoS One. 2017 Oct 10;12(10):e0185943. doi: 10.1371/journal.pone.0185943 (PMC5634593; doi:10.1371/journal.pone.0185943)

S5 Figure.

Nitric Oxide Signaling

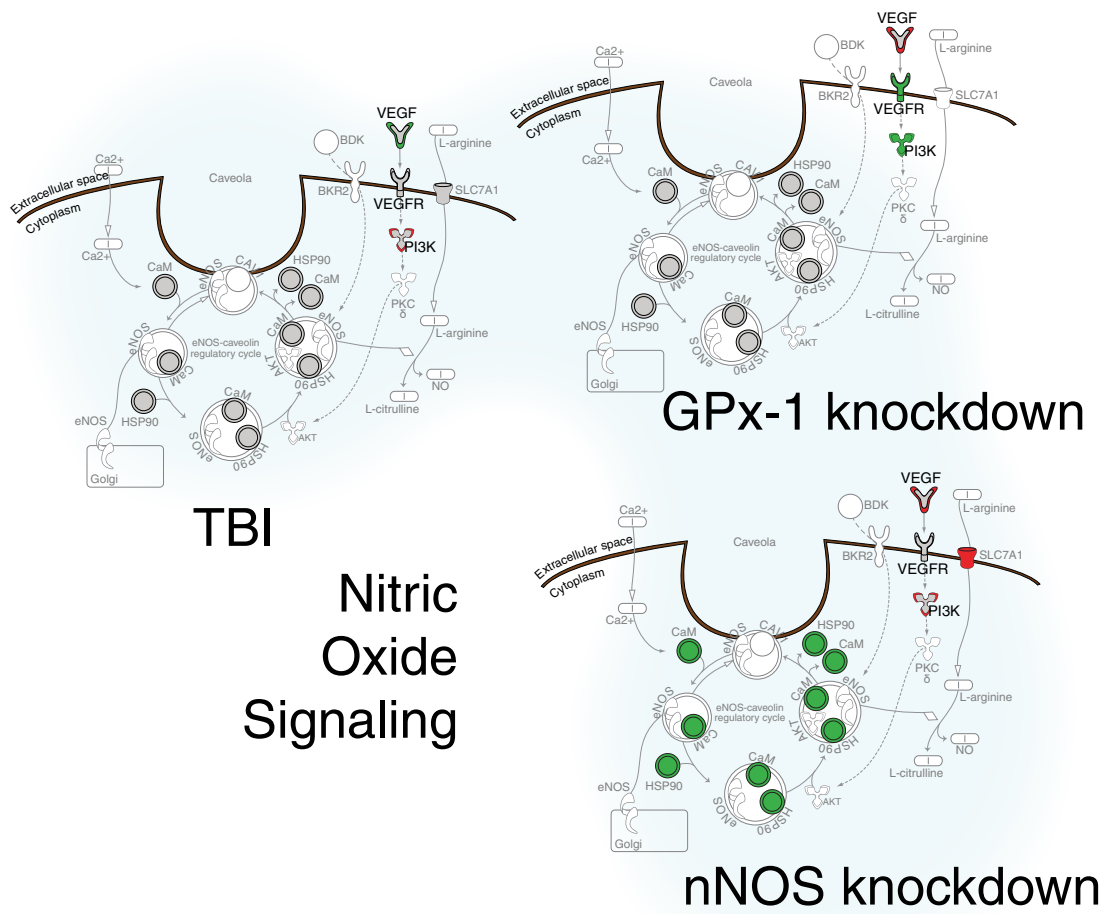

TBI

Nitric  
Oxide  
Signaling

GPx-1 knockdown

nNOS knockdown

Supplement: S5 Fig — (PDF) [file pone.0185943.s005.pdf]

## Enlargement of Fig 7A

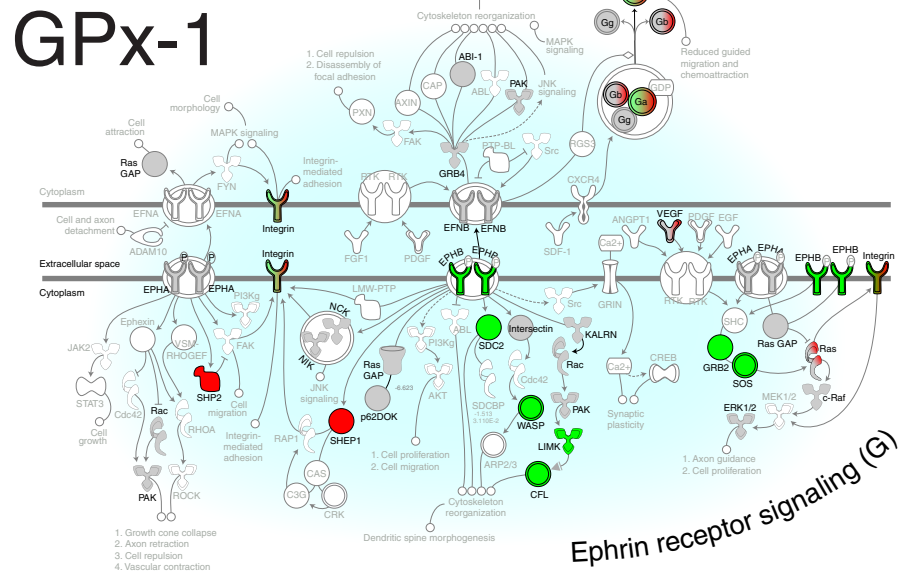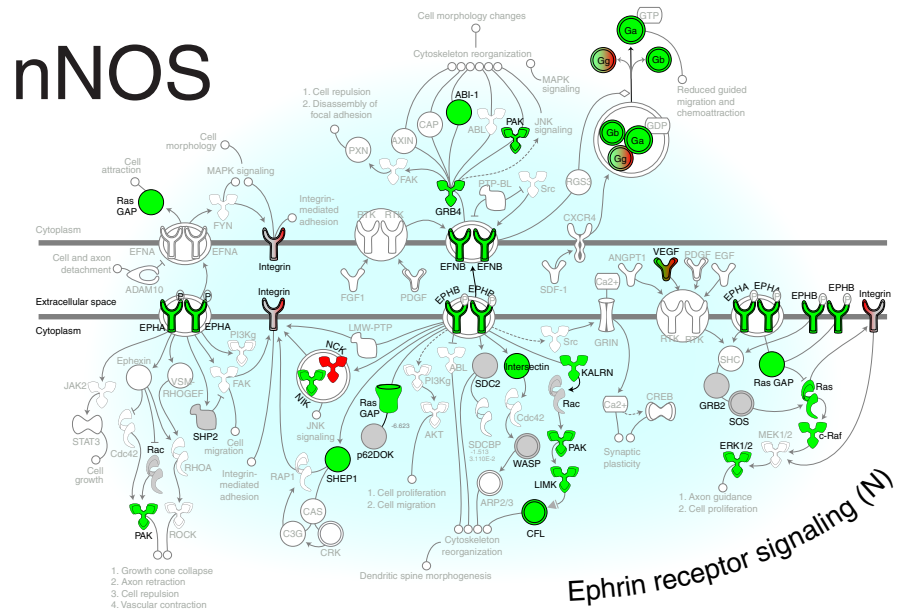

Supplement: S6 Fig — (PDF) [file pone.0185943.s006.pdf]

## Enlargement of Fig 7B

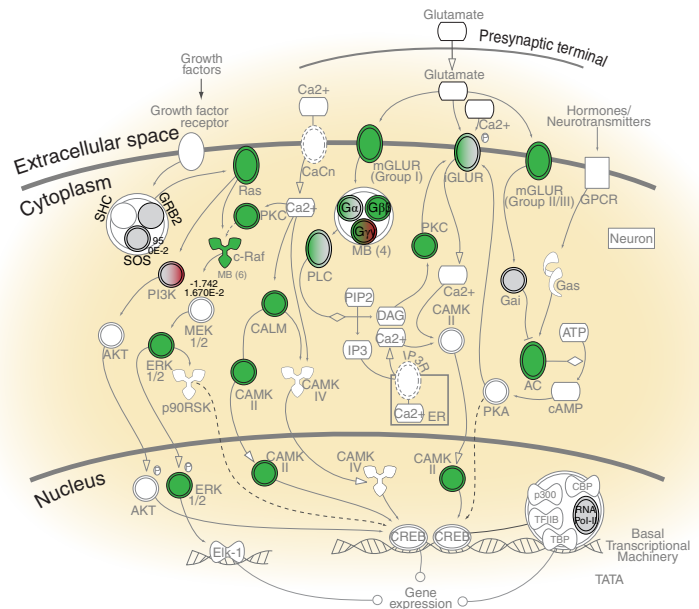

Supplement: S7 Fig — (PDF) [file pone.0185943.s007.pdf]
