# Supplementary material for: Effects of AAV-mediated knockdown of nNOS and GPx-1 gene expression in rat hippocampus after traumatic brain injury
Source: PLoS One. 2017 Oct 10;12(10):e0185943. doi: 10.1371/journal.pone.0185943 (PMC5634593; doi:10.1371/journal.pone.0185943)

S8 Figure. GABA signaling pathway differentiall affected by nNOS or GPx knockdown

Enlargement of Fig 7C

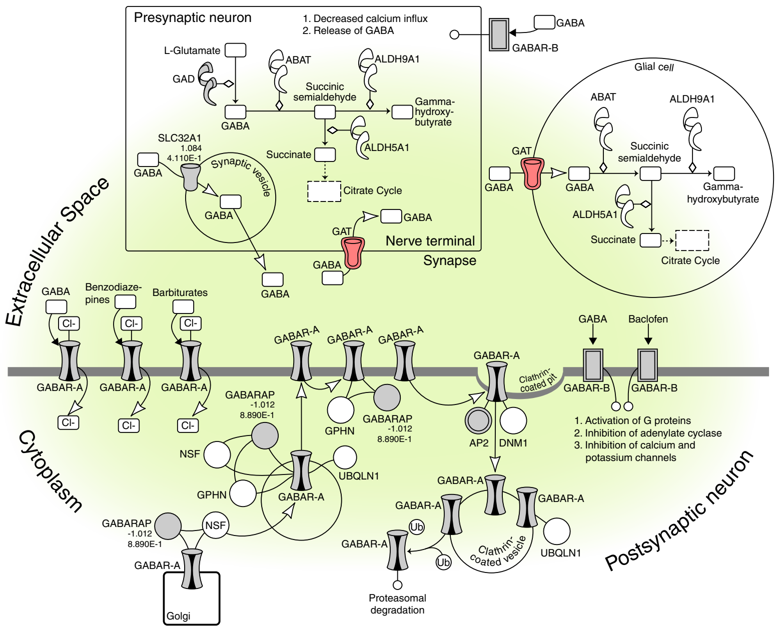

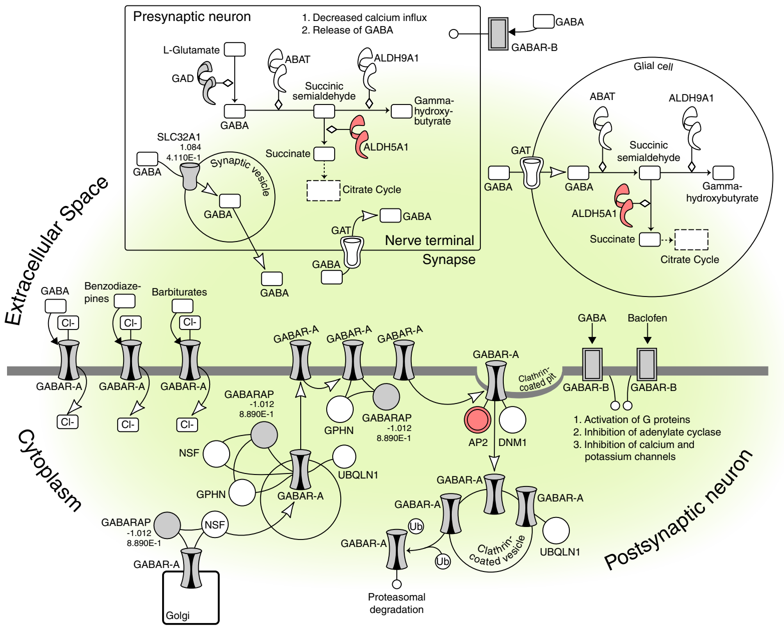

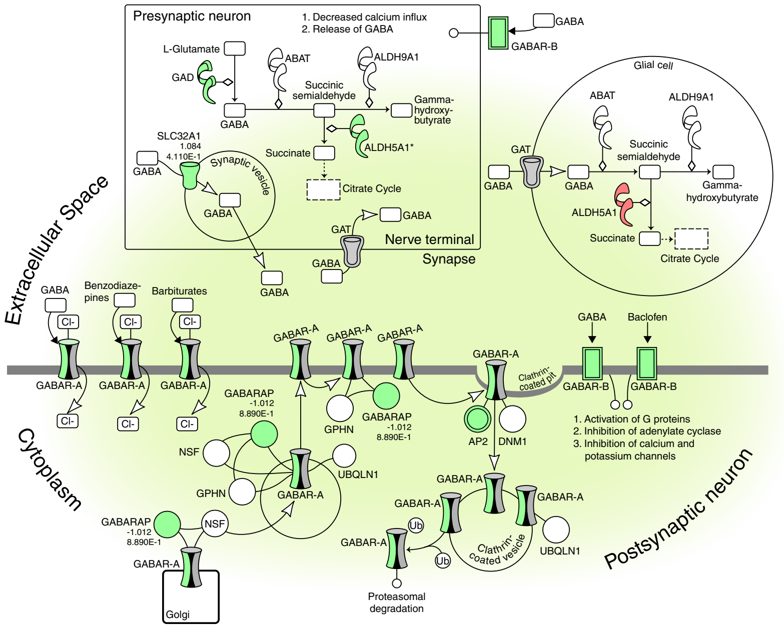

Supplement: S8 Fig — (PDF) [file pone.0185943.s008.pdf]
